# Crystal structure, Hirshfeld surface analysis and DFT studies of 6-bromo-3-(12-bromo­dodec­yl)-2-(4-nitro­phen­yl)-4*H*-imidazo[4,5-*b*]pyridine

**DOI:** 10.1107/S2056989020005228

**Published:** 2020-04-21

**Authors:** Zainab Jabri, Karim Jarmoni, Tuncer Hökelek, Joel T. Mague, Safia Sabir, Youssef Kandri Rodi, Khalid Misbahi

**Affiliations:** a Laboratory of Applied Organic Chemistry, Sidi Mohamed Ben Abdellah University, Faculty of Sciences and Techniques, Road Immouzer, BP 2202 Fez, Morocco; b Department of Physics, Hacettepe University, 06800 Beytepe, Ankara, Turkey; c Department of Chemistry, Tulane University, New Orleans, LA 70118, USA

**Keywords:** crystal structure, imidazole, pyridine, π-stacking, hydrogen bond, disorder

## Abstract

The title compound, C_24_H_30_Br_2_N_4_O_2_, consists of a 2-(4-nitro­phen­yl)-4*H*-imidazo[4,5-*b*]pyridine entity with a 12-bromo­dodecyl substituent attached to the pyridine N atom. The middle eight-carbon portion of the side chain is planar to within 0.09 (1) Å and makes a dihedral angle of 21.9 (8)° with the mean plane of the imidazolo­pyridine moiety, giving the mol­ecule a V-shape. In the crystal, the imidazolo­pyridine units are associated through slipped π–π stacking inter­actions together with weak C—H_Pyr_⋯O_Ntr_ and C—H_Brmdc­yl_⋯O_Ntr_ (Pyr = pyridine, Ntr = nitro and Brmdcyl = bromo­dodec­yl) hydrogen bonds. The 12-bromo­dodecyl chains overlap with each other between the stacks. The terminal –CH_2_Br group of the side chain shows disorder over two resolved sites in a 0.902 (3):0.098 (3) ratio. Hirshfeld surface analysis indicates that the most important contributions for the crystal packing are from H⋯H (48.1%), H⋯Br/Br⋯H (15.0%) and H⋯O/O⋯H (12.8%) inter­actions. The optimized mol­ecular structure, using density functional theory at the B3LYP/ 6–311 G(d,p) level, is compared with the experimentally determined structure in the solid state. The HOMO–LUMO behaviour was elucidated to determine the energy gap.

## Abstract 2

In the title mol­ecule, the imidazolo­pyridine and phenyl rings are nearly co-planar, and the 12-bromo­dodecyl side chain is directed so as to give the complete mol­ecule a V-shape.

## Chemical context   

Imidazole derivatives are a class of heterocyclic compounds exhibiting pharmacological activities across a wide range of therapeutic targets (El Kazzouli *et al.*, 2011 ▸; Martínez-Urbina *et al.*, 2010 ▸). Imidazo­pyridine derivatives are used in medicinal chemistry because of their biological and pharmacological properties, in particular as anti-inflammatory, anti-cancer, anti­viral, anti-osteoporotic, anti-parasitic and anti­hypertensive agents. Certain compounds with an imidazo­pyridine skeleton are used to treat psychiatric and autoimmune disorders (Dymínska, 2015 ▸; Bababdani & Mousavi, 2013 ▸). They have also been approved as effective anti­fungal agents and exhibit anti­microbial activities (Devi *et al.*, 2018 ▸), some of which can be used for the treatment of disorders characterized by activation of the Wnt signaling pathway (cancer, abnormal cell proliferation, angiogenesis, fibrous disorders, bone or cartilage and arthritis), as well as genetic and neurological diseases. On the other hand, imidazo­pyridine compounds, which have a specificity to GPR4 as negative allosteric modulators (Tobo *et al.*, 2015 ▸), can also be used for the treatment of gastric and/or duodenal ulcers. Several imidazo[4,5-*b*]pyridine derivatives have also been reported as corrosion inhibitors of steel in an acidic environment (Bouayad *et al.*, 2018 ▸; Sikine *et al.*, 2016 ▸; Yadav *et al.*, 2014 ▸), and also as inhibitors of a nanomolar rhodesaine (Ehmke *et al.*, 2013 ▸) or the Et-PKG enzyme (Cheng *et al.*, 2010 ▸).
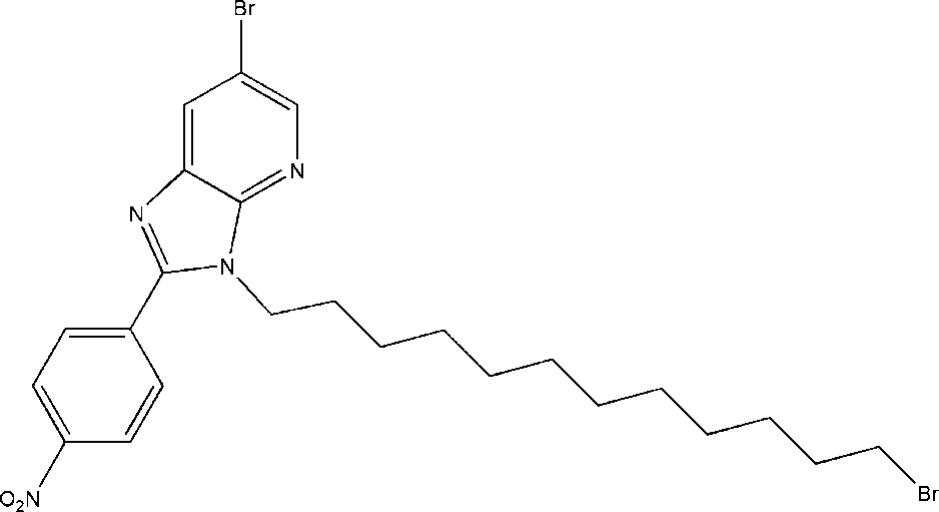


Following our research work directed at obtaining new heterocyclic compounds having an imidazo[4,5-*b*]pyridine moiety, we were inter­ested in the condensation of imidazo[4,5-*b*]pyridine derivatives with di-halogenated chains under phase-transfer catalysis (PTC) conditions. We report herein the synthesis and the mol­ecular and crystal structures of the title compound (I) together with Hirshfeld surface analysis and DFT calculations for comparison with the experimentally determined mol­ecular structure in the solid state.

## Structural commentary   

**Figure 1:**
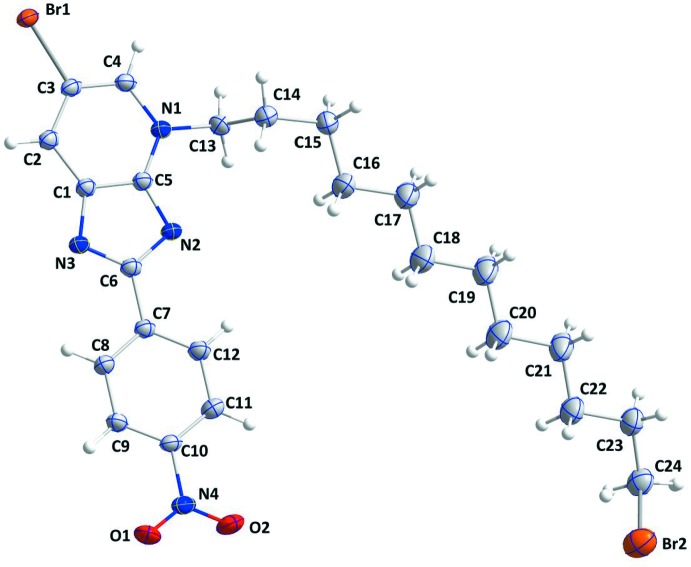
The asymmetric unit of (I) with the atom-numbering scheme. Displacement ellipsoids are drawn at the 50% probability level. Only the major part of the disordered –CH_2_Br group is shown.

The mol­ecule of (I) consists of an imidazolo­pyridine moiety to which a nitro­phenyl group is attached to the C atom (C6) of the five-membered ring. The 12-bromo­dodecyl side chain is attached to one of the two N atoms (N1). The imidazolo­pyridine moiety is planar to within 0.0208 (15) Å (r.m.s. deviation = 0.0122 Å) with atom C3 being the most distant from the least-squares plane. The phenyl ring (C7–C12) is inclined to the above plane by only 1.6 (1)°, making the two ring systems essentially planar. The C4—N1—C13—C14 torsion angle (C13 and C14 are the first two atoms of the 12-bromo­dodecyl chain) is 95.7 (2)°. The C15–C22 portion of the 12-bromo­dodecyl chain is approximately planar [maximum deviation from the least-squares plane running through the eight C atoms is 0.09 (1) Å for C20], and this plane is inclined to that of the imidazolo­pyridine moiety by 21.9 (8)°, giving the mol­ecule an overall V-shape (Fig. 1 ▸). The terminal C24—Br2 portion of the 12-bromo­dodecyl chain is disordered over two resolved sites in a refined ratio of 0.902 (3):0.098 (3).

## Supra­molecular features   

**Figure 2:**
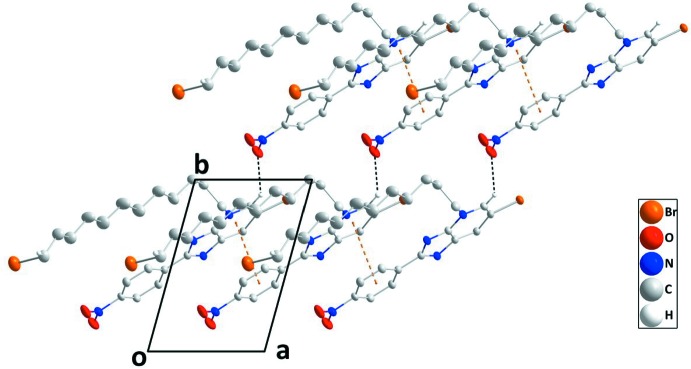
Detail of the inter­molecular inter­actions viewed along the *c-*axis direction. The weak C—H_Pyr_⋯O_Ntr_ and C—H_Brmdc­yl_⋯O_Ntr_ (Pyr = pyridine, Ntr = nitro and Brmdcyl = bromo­dodec­yl) hydrogen bonds and π–π stacking inter­actions are depicted, respectively, by black and orange dashed lines.

**Figure 3:**
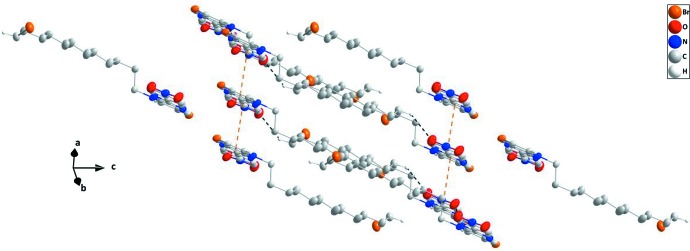
A partial packing diagram projected onto (581) with inter­molecular inter­actions depicted as in Fig. 2 ▸.

In the crystal, sloped stacks of mol­ecules extending along the *a-*axis direction are formed by slipped π–π stacking inter­actions between the N1/C1–C5 and C7–C12 rings, with a centroid-to-centroid distance of 3.5308 (13) Å, a dihedral angle of 1.83 (9)° and a slippage of 1.192 Å. The angle between the plane defined by the relevant centroids in the stack and (001) is 19.210 (11)°. The stacks are connected by weak C—H_Pyr_⋯O_Ntr_ (Pyr = pyridine and Ntr = nitro) hydrogen bonds (Table 1 ▸, Fig. 2 ▸). Additional linkage is accomplished by C—H_Brmdc­yl_⋯O_Ntr_ (Brmdcyl = bromo­dodec­yl) hydrogen bonds that influence the arrangement of the 12-bromo­dodecyl chains between adjacent stacks (Table 1 ▸, Fig. 3 ▸).

## Hirshfeld surface analysis   

**Figure 4:**
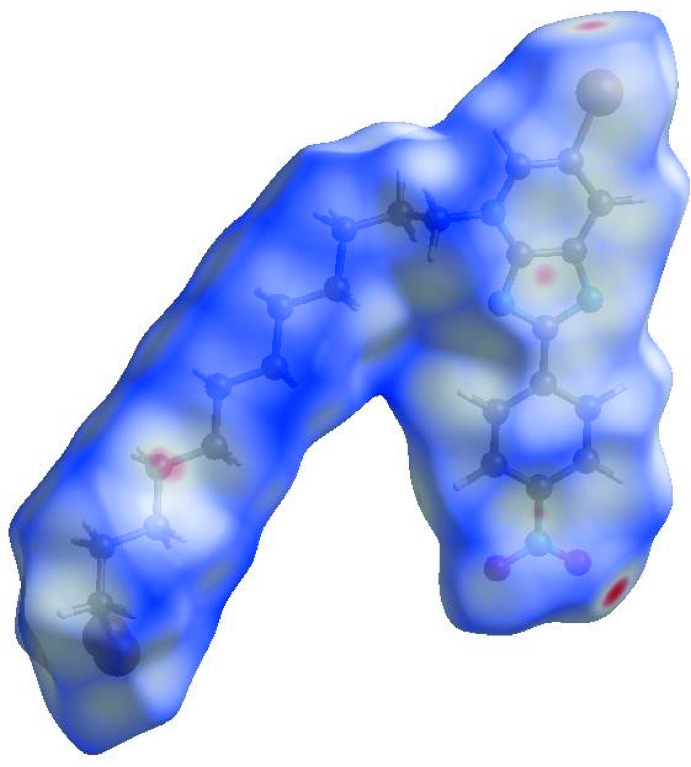
View of the three-dimensional Hirshfeld surface of the title compound plotted over *d*_norm_ in the range −0.1956 to 1.3971 a.u..

**Figure 5:**
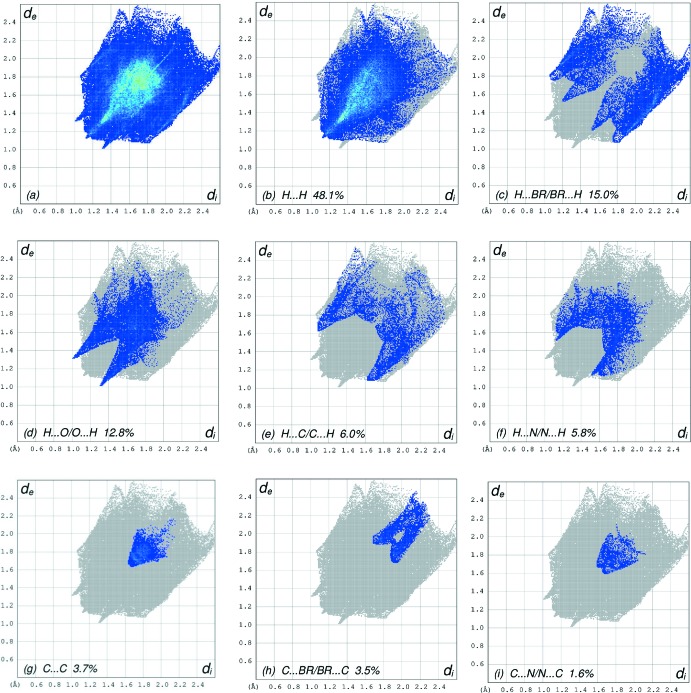
Two-dimensional fingerprint plots for (I), showing (*a*) all inter­actions, and delineated into (*b*) H⋯H, (*c*) H⋯Br/Br⋯H, (*d*) H⋯O/O⋯H, (*e*) H⋯C/C⋯H, (*f*) H ⋯N/N⋯H, (*g*) C⋯C, (*h*) C⋯Br/Br⋯C and (i) C⋯N/N⋯C inter­actions. The *d*_i_ and *d*_e_ values are the closest inter­nal and external distances (in Å) from given points on the Hirshfeld surface contacts.

In order to qu­antify and visualize the inter­molecular inter­actions in the crystal of (I), a Hirshfeld surface (HS) analysis (Hirshfeld, 1977 ▸; Spackman & Jayatilaka, 2009 ▸) was carried out by using *Crystal Explorer 17.5* (Turner *et al.*, 2017 ▸). In the HS plotted over *d*_norm_ (Fig. 4 ▸), the white surface indicates contacts with distances equal to the sum of van der Waals radii, and the red and blue colours indicate distances shorter (in close contact) or longer (distant contact) than the van der Waals radii, respectively (Venkatesan *et al.*, 2016 ▸). The overall two-dimensional fingerprint plot, Fig. 5 ▸ *a*, and those delineated into H⋯H, H⋯Br/Br⋯H, H⋯O/O⋯H, H⋯C/C⋯H, H⋯N/N⋯H, C⋯C, C⋯Br/Br⋯C and C⋯N/N⋯C contacts (McKinnon *et al.*, 2007 ▸) are illustrated in Fig. 5 ▸ *b*–*i*, respectively, together with their relative contributions to the HS. The large number of these contacts suggests that van der Waals inter­actions and hydrogen bonding play the major roles in the crystal packing (Hathwar *et al.*, 2015 ▸). The most important inter­action is H⋯H contributing 48.1% to the overall crystal packing, Fig. 5 ▸ *b*, followed by H⋯Br/Br⋯H, Fig. 5 ▸ *c*, contacts at 15.0%, H⋯O/O⋯H, Fig. 5 ▸ *d*, contacts at 12.8%, H⋯C/C⋯H, Fig. 5 ▸ *e*, contacts at 6.0%, H⋯/N⋯H, Fig. 5 ▸ *f*, contacts at 5.8%, C⋯C, Fig. 5 ▸ *g* contacts at 3.7%, C⋯Br/Br⋯C, Fig. 5 ▸ *h*, contacts at 3.5%, and C⋯N/N⋯C, Fig. 5 ▸ *i*, contacts at 1.6%.

## DFT calculations   

**Figure 6:**
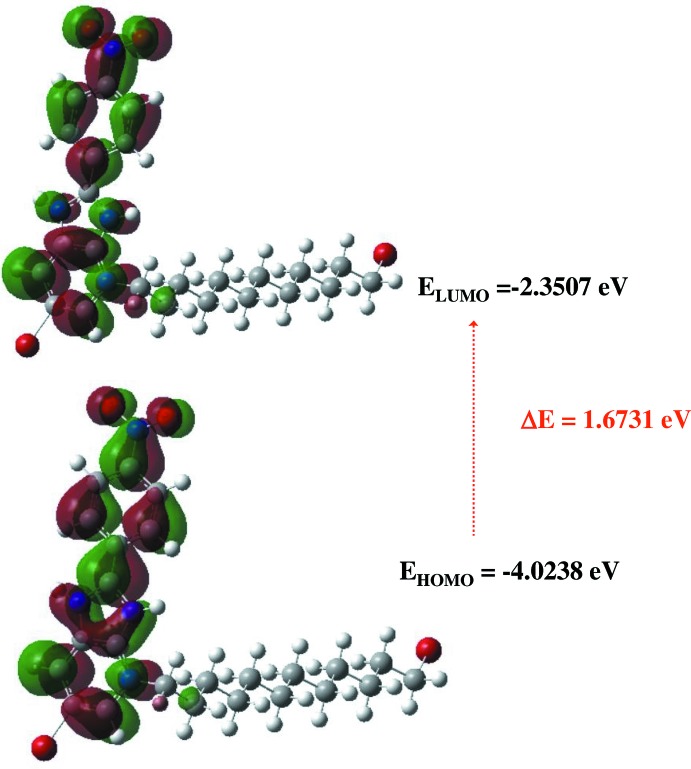
Shapes of HOMO and LUMO of (I) and the energy band gap between them.

The optimized structure of the mol­ecule in the gas phase was calculated *via* density functional theory (DFT) using the standard B3LYP functional and 6–311G(d,p) basis-set calculations (Becke, 1993 ▸) as implemented in *GAUSSIAN 09* (Frisch *et al.*, 2009 ▸). The theoretical and experimental bond lengths and angles are in good agreement (Table 2 ▸). Results for *E*_HOMO_ and *E*_LUMO_ energies, electronegativity (χ), hardness (η), potential (μ), electrophilicity (ω) and softness (*σ*) are collated in Table 3 ▸. The electron transition from the HOMO to the LUMO energy level is shown in Fig. 6 ▸. The energy band gap [Δ*E* = *E*_LUMO_ – *E*_HOMO_] of the mol­ecule is 1.6731 eV, and the calculated frontier mol­ecular orbital energies, *E*_HOMO_ and *E*_LUMO_, are −4.0238 and −2.3507 eV, respectively.

## Database survey   

**Figure 7:**
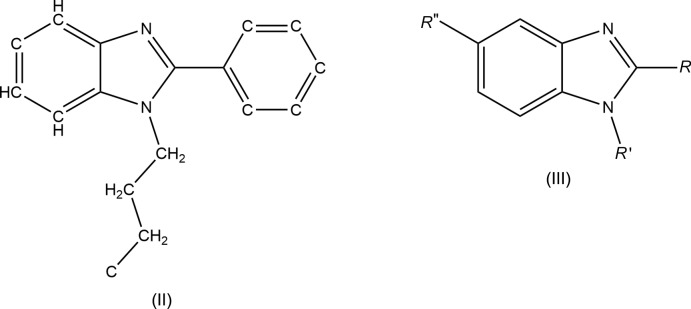
Lewis structures of fragments (II) and (III) used in the database search.

The importance of benzimidazole derivatives is highlighted by a search of the Cambridge Structural Database (CSD, updated to November 2019; Groom *et al.*, 2016 ▸) using a fragment allowing for substituents at the 6-position and having a single carbon atom at the 3- and 4-positions, which resulted in over 1900 hits. Restricting the search to exclude metal complexes and using the fragment (II) (Fig. 7 ▸) yielded eight hits most comparable to (I). These are of the general type (III) (Fig. 7 ▸) with *R*′′ = H, *R* = 4-[Ph_2_C=C(Ph)]C_6_H_4_, *R*′ = *n*-Bu (VEDKEX; Zhang *et al.*, 2018 ▸), *n*-hexyl (VEDHUK; Zhang *et al.*, 2018 ▸) and *R*′ = 6-(9*H*-carbazol-9-yl)hexyl, *R* = Ph (DUKJAV; Zhao *et al.*, 2009 ▸). Additional ones have *R*′′ = COOMe, *R*′ = *n*-Bu, *R* = 3,4-Cl_2_C_6_H_3_ (ABEJAT; Arslan *et al.*, 2004 ▸), 2-[2-(cinnamyl­thio)­benzo[*d*]oxazol-5-yl (TAPVIR; Chanda *et al.*, 2012 ▸), and the set is completed by those with *R*′ = *n*-Bu, *R*′′ = CN, *R* = 3-ClC_6_H_4_ (WEWVIE; Kazak *et al.*, 2006 ▸) or *R*= 3,4-(MeO)_2_C_6_H_3_ (WEWVOK; Kazak *et al.*, 2006 ▸) and *R*′′ = NO_2_, *R*′ = *n*-Bu, *R* = 2-(*p*TosNH)C_6_H_4_ (BUXDUV; Burlov *et al.*, 2016 ▸). In all of the matches where *R*′ is an alkyl chain, the base of the chain is approximately perpendicular to the benzimidazole plane but not all of them have the remainder of the chain in a fully extended conformation and in no instances are structures seen in which the alkyl chains overlap. Part of the reason is that the butyl group is not long enough to counteract the packing inter­actions involving the benzimidazole moiety and the substituents in the 2-position. The possible exception is in DUKJAV where π–π stacking inter­actions appear to occur between the benzimidazole units and between the carbazole units. Also, all of the related mol­ecules identified have a dihedral angle between the plane of the benzimidazole unit and the plane of the aromatic ring in the 2-position of 28–48° while in (I) the two are nearly coplanar [1.6 (1)°]. This is likely due to packing considerations.

## Synthesis and crystallization   

To a solution of 6-bromo-2-(4′-nitro­phen­yl)-3*H*-imidazo[4,5-*b*]pyridine (0.4 g, 1.25 mmol), potassium carbonate (2.2 equivalents; 0.38 g, 2.75 mmol) and tetra-*n*-butyl­ammonium bromide (0.2 equivalents; 0.061 g, 0.187 mmo)l in 40 ml of DMF were added in small portions to 1.5 equivalents of 1,12-di­bromo­dodecane. The resulting mixture was stirred magnetically at room temperature for 48 h. After removal of the salts and evaporation of DMF under reduced pressure, the product was separated by chromatography on a column of silica gel using a mixture of hexa­ne/di­chloro­methane: 1/4 (*v*/*v*) as the mobile phase. Orange single crystals suitable for X-ray diffraction were obtained by slow evaporation of the eluant.

## Refinement   

Crystal, data collection and refinement details are presented in Table 4 ▸. Hydrogen atoms were included as riding contributions in idealized positions with isotropic displacement parameters tied to those of the attached atoms. The terminal C24—Br2 portion of the 12-bromo­dodecyl chain is disordered over two resolved sites in a 0.902 (3)/0.098 (3) ratio. The two components of the disorder were refined with restraints so that their bond lengths and angles are comparable.

## Supplementary Material

Crystal structure: contains datablock(s) I, global. DOI: 10.1107/S2056989020005228/wm5551sup1.cif

Supporting information file. DOI: 10.1107/S2056989020005228/wm5551Isup3.cdx

Structure factors: contains datablock(s) I. DOI: 10.1107/S2056989020005228/wm5551Isup4.hkl

Supporting information file. DOI: 10.1107/S2056989020005228/wm5551Isup4.cml

CCDC reference: 1996788

Additional supporting information:  crystallographic information; 3D view; checkCIF report

## Figures and Tables

**Table 1:** Hydrogen-bond geometry (Å, °)

*D*—H⋯*A*	*D*—H	H⋯*A*	*D*⋯*A*	*D*—H⋯*A*
C4—H4⋯O1^i^	0.95	2.43	3.171 (3)	135
C24—H24*A*⋯O2^ii^	0.99	2.53	3.372 (3)	142

Symmetry codes: (i) 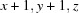; (ii) 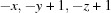.

**Table 2:** Comparison of selected (X-ray and DFT) geometric data (Å, °) for (I)

Bonds/angles	X-ray	B3LYP/6–311G(d,p)
Br1—C1	1.8954 (19)	1.94573
Br2—C24	1.953 (3)	2.02642
O1—N4	1.224 (2)	1.28476
O2—N4	1.233 (2)	1.28545
N1—C5	1.357 (2)	1.36426
N1—C4	1.361 (2)	1.42977
N1—C13	1.473 (3)	1.47563
N2—C5	1.336 (2)	1.35689
N2—C6	1.372 (3)	1.40945
N3—C6	1.346 (2)	1.40939
N3—C1	1.372 (2)	1.38222
N4—C10	1.465 (2)	1.42118
C5—N1—C4	118.57 (17)	117.37
C5—N1—C13	119.67 (16)	119.58
C4—N1—C13	121.75 (17)	120.96
C5—N2—C6	100.79 (16)	101.37
C6—N3—C1	102.17 (16)	101.96
O1—N4—O2	123.30 (19)	122.47
O1—N4—C10	118.48 (18)	118.78
O2—N4—C10	118.22 (18)	118.74
N3—C1—C2	132.04 (18)	133.14
N3—C1—C5	107.59 (16)	105.38
C2—C1—C5	120.37 (18)	121.46

**Table 3:** Calculated mol­ecular energies for (I)

Total Energy, *TE* (eV)	−175539.349
*E*_HOMO_ (eV)	−4.0238
*E*_LUMO_ (eV)	−2.3507
Gap, *ΔE* (eV)	1.6731
Dipole moment, *μ* (Debye)	15.7142
Ionization potential, *I* (eV)	4.0238
Electron affinity, *A*	2.3507
Electronegativity, *χ*	3.1872
Hardness, *η*	0.8366
Electrophilicity index, *ω*	3.0715
Softness, *σ*	1.1954
Fraction of electron transferred, *ΔN*	2.2788

**Table 4:** Experimental details

Crystal data	
Chemical formula	C_24_H_30_Br_2_N_4_O_2_
*M*_r_	566.34
Crystal system, space group	Triclinic, *P* 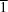
Temperature (K)	150
*a*, *b*, *c* (Å)	6.3291 (11), 9.8911 (17), 21.133 (4)
α, β, γ (°)	76.480 (2), 84.965 (3), 73.891 (2)
*V* (Å^3^)	1235.4 (4)
*Z*	2
Radiation type	Mo *K*α
μ (mm^−1^)	3.31
Crystal size (mm)	0.48 × 0.23 × 0.20

Data collection	
Diffractometer	Bruker SMART APEX CCD
Absorption correction	Multi-scan (*SADABS*; Krause *et al.*, 2015 ▸)
*T*_min_, *T*_max_	0.23, 0.56
No. of measured, independent and observed [*I* > 2σ(*I*)] reflections	24139, 6598, 5337
*R*_int_	0.040
(sin θ/λ)_max_ (Å^−1^)	0.688

Refinement	
*R*[*F* ^2^ > 2σ(*F* ^2^)], *wR*(*F* ^2^), *S*	0.035, 0.095, 1.03
No. of reflections	6598
No. of parameters	293
H-atom treatment	H-atom parameters constrained
Δρ_max_, Δρ_min_ (e Å^−3^)	0.72, −0.80

Computer programs: *APEX3* and *SAINT* (Bruker, 2016 ▸), *SHELXT* (Sheldrick, 2015*a* ▸), *SHELXL2018/1* (Sheldrick, 2015*b* ▸), *DIAMOND* (Brandenburg & Putz, 2012 ▸) and *publCIF* (Westrip, 2010 ▸).

## supplementary crystallographic information

### Crystal data

**Table:**

C_24_H_30_Br_2_N_4_O_2_	*Z* = 2
*M**_r_* = 566.34	*F*(000) = 576
Triclinic, *P*1	*D*_x_ = 1.522 Mg m^−^^3^
*a* = 6.3291 (11) Å	Mo *K*α radiation, λ = 0.71073 Å
*b* = 9.8911 (17) Å	Cell parameters from 9900 reflections
*c* = 21.133 (4) Å	θ = 2.2–29.0°
α = 76.480 (2)°	µ = 3.31 mm^−^^1^
β = 84.965 (3)°	*T* = 150 K
γ = 73.891 (2)°	Column, light orange
*V* = 1235.4 (4) Å^3^	0.48 × 0.23 × 0.20 mm

### Data collection

**Table:**

Bruker SMART APEX CCD diffractometer	6598 independent reflections
Radiation source: fine-focus sealed tube	5337 reflections with *I* > 2σ(*I*)
Graphite monochromator	*R*_int_ = 0.040
Detector resolution: 8.3333 pixels mm^-1^	θ_max_ = 29.3°, θ_min_ = 2.0°
φ and ω scans	*h* = −8→8
Absorption correction: multi-scan (*SADABS*; Krause *et al.*, 2015)	*k* = −13→13
*T*_min_ = 0.23, *T*_max_ = 0.56	*l* = −29→29
24139 measured reflections	

### Refinement

**Table:**

Refinement on *F*^2^	Primary atom site location: dual
Least-squares matrix: full	Secondary atom site location: difference Fourier map
*R*[*F*^2^ > 2σ(*F*^2^)] = 0.035	Hydrogen site location: inferred from neighbouring sites
*wR*(*F*^2^) = 0.095	H-atom parameters constrained
*S* = 1.03	*w* = 1/[σ^2^(*F*_o_^2^) + (0.0494*P*)^2^ + 0.370*P*] where *P* = (*F*_o_^2^ + 2*F*_c_^2^)/3
6598 reflections	(Δ/σ)_max_ = 0.002
293 parameters	Δρ_max_ = 0.72 e Å^−^^3^
0 restraints	Δρ_min_ = −0.80 e Å^−^^3^

### Special details

**Table:**

Experimental. The diffraction data were obtained from 3 sets of 400 frames, each of width 0.5° in ω, colllected at φ = 0.00, 90.00 and 180.00° and 2 sets of 800 frames, each of width 0.45° in φ, collected at ω = -30.00 and 210.00°. The scan time was 10 sec/frame.
Geometry. All esds (except the esd in the dihedral angle between two l.s. planes) are estimated using the full covariance matrix. The cell esds are taken into account individually in the estimation of esds in distances, angles and torsion angles; correlations between esds in cell parameters are only used when they are defined by crystal symmetry. An approximate (isotropic) treatment of cell esds is used for estimating esds involving l.s. planes.
Refinement. Refinement of F^2^ against ALL reflections. The weighted R-factor wR and goodness of fit S are based on F^2^, conventional R-factors R are based on F, with F set to zero for negative F^2^. The threshold expression of F^2^ > 2sigma(F^2^) is used only for calculating R-factors(gt) etc. and is not relevant to the choice of reflections for refinement. R-factors based on F^2^ are statistically about twice as large as those based on F, and R- factors based on ALL data will be even larger. H-atoms attached to carbon were placed in calculated positions (C—H = 0.95 - 0.99 Å). All were included as riding contributions with isotropic displacement parameters 1.2 - 1.5 times those of the attached atoms. Br2 is disordered over two sites in a 0.902 (3)/0.098 (32) ratio with the two components refined with restraints that they have comparable geometries.

### Fractional atomic coordinates and isotropic or equivalent isotropic displacement parameters (Å^2^)

**Table:**

	*x*	*y*	*z*	*U*_iso_*/*U*_eq_	Occ. (<1)
Br1	1.82823 (3)	0.88087 (2)	0.00828 (2)	0.02928 (7)	
Br2	−0.34993 (9)	0.51084 (14)	0.63610 (3)	0.05439 (19)	0.902 (3)
Br2A	−0.3773 (9)	0.5733 (11)	0.6273 (3)	0.05439 (19)	0.098 (3)
O1	0.4691 (3)	0.16270 (19)	0.13274 (10)	0.0470 (4)	
O2	0.3734 (3)	0.2314 (2)	0.22339 (9)	0.0468 (4)	
N1	1.3755 (3)	0.79656 (18)	0.15128 (8)	0.0244 (3)	
N2	1.1281 (3)	0.64593 (18)	0.16156 (8)	0.0239 (3)	
N3	1.2508 (3)	0.55633 (18)	0.06847 (8)	0.0234 (3)	
N4	0.4845 (3)	0.23168 (19)	0.17246 (10)	0.0317 (4)	
C1	1.3664 (3)	0.6514 (2)	0.07409 (9)	0.0222 (4)	
C2	1.5326 (3)	0.6991 (2)	0.03550 (10)	0.0241 (4)	
H2	1.588946	0.665956	−0.002911	0.029*	
C3	1.6108 (3)	0.7989 (2)	0.05705 (10)	0.0241 (4)	
C4	1.5370 (3)	0.8446 (2)	0.11371 (10)	0.0250 (4)	
H4	1.599544	0.910484	0.126734	0.030*	
C5	1.2880 (3)	0.7032 (2)	0.13151 (9)	0.0223 (4)	
C6	1.1134 (3)	0.5596 (2)	0.12076 (9)	0.0225 (4)	
C7	0.9500 (3)	0.4757 (2)	0.13404 (9)	0.0223 (4)	
C8	0.9326 (3)	0.3904 (2)	0.09185 (10)	0.0256 (4)	
H8	1.027479	0.386290	0.054538	0.031*	
C9	0.7781 (3)	0.3117 (2)	0.10385 (10)	0.0266 (4)	
H9	0.764906	0.253965	0.074998	0.032*	
C10	0.6431 (3)	0.3189 (2)	0.15897 (10)	0.0249 (4)	
C11	0.6535 (3)	0.4038 (2)	0.20136 (10)	0.0272 (4)	
H11	0.557276	0.407835	0.238384	0.033*	
C12	0.8083 (3)	0.4831 (2)	0.18856 (10)	0.0254 (4)	
H12	0.817963	0.542629	0.216954	0.031*	
C13	1.2921 (3)	0.8451 (2)	0.21174 (10)	0.0277 (4)	
H13A	1.407583	0.875235	0.229259	0.033*	
H13B	1.259112	0.763605	0.244503	0.033*	
C14	1.0856 (4)	0.9702 (2)	0.20091 (10)	0.0303 (4)	
H14A	0.979474	0.946902	0.176234	0.036*	
H14B	1.124005	1.057448	0.174668	0.036*	
C15	0.9786 (4)	1.0010 (2)	0.26569 (11)	0.0354 (5)	
H15A	1.093787	1.003377	0.293931	0.042*	
H15B	0.872569	1.097574	0.257308	0.042*	
C16	0.8594 (4)	0.8905 (3)	0.30167 (12)	0.0422 (6)	
H16A	0.766800	0.873698	0.270434	0.051*	
H16B	0.970288	0.798211	0.317524	0.051*	
C17	0.7154 (5)	0.9319 (3)	0.35888 (13)	0.0481 (7)	
H17A	0.608784	1.026430	0.343710	0.058*	
H17B	0.808726	0.943166	0.391508	0.058*	
C18	0.5895 (5)	0.8219 (3)	0.39151 (13)	0.0498 (7)	
H18A	0.499382	0.809373	0.358399	0.060*	
H18B	0.697061	0.727992	0.407038	0.060*	
C19	0.4406 (5)	0.8604 (3)	0.44815 (14)	0.0551 (8)	
H19A	0.334976	0.955515	0.433229	0.066*	
H19B	0.530537	0.869341	0.482227	0.066*	
C20	0.3130 (5)	0.7502 (3)	0.47773 (13)	0.0511 (7)	
H20A	0.418487	0.653915	0.489418	0.061*	
H20B	0.215232	0.746721	0.444409	0.061*	
C21	0.1746 (5)	0.7810 (3)	0.53790 (14)	0.0504 (7)	
H21A	0.268887	0.794690	0.569570	0.060*	
H21B	0.057909	0.872000	0.525371	0.060*	
C22	0.0687 (5)	0.6614 (3)	0.57043 (12)	0.0434 (6)	
H22A	0.182702	0.568038	0.577361	0.052*	
H22B	−0.041627	0.656911	0.541078	0.052*	
C23	−0.0432 (5)	0.6828 (3)	0.63564 (12)	0.0434 (6)	
H23A	0.059462	0.705909	0.661635	0.052*	
H23B	−0.173900	0.766726	0.627551	0.052*	
C24	−0.1145 (4)	0.5542 (3)	0.67503 (11)	0.0404 (5)	0.902 (3)
H24A	−0.164784	0.571989	0.718783	0.049*	0.902 (3)
H24B	0.014190	0.468801	0.680713	0.049*	0.902 (3)
C24A	−0.1145 (4)	0.5542 (3)	0.67503 (11)	0.0404 (5)	0.098 (3)
H24C	−0.150774	0.561926	0.720811	0.049*	0.098 (3)
H24D	−0.001810	0.462587	0.673847	0.049*	0.098 (3)

### Atomic displacement parameters (Å^2^)

**Table:**

	*U*^11^	*U*^22^	*U*^33^	*U*^12^	*U*^13^	*U*^23^
Br1	0.02318 (11)	0.02607 (11)	0.04000 (13)	−0.01178 (8)	0.00655 (8)	−0.00638 (8)
Br2	0.0548 (2)	0.0723 (6)	0.04320 (19)	−0.0293 (3)	−0.00326 (15)	−0.0109 (3)
Br2A	0.0548 (2)	0.0723 (6)	0.04320 (19)	−0.0293 (3)	−0.00326 (15)	−0.0109 (3)
O1	0.0496 (11)	0.0440 (10)	0.0645 (12)	−0.0309 (8)	0.0154 (9)	−0.0294 (9)
O2	0.0465 (10)	0.0549 (11)	0.0518 (11)	−0.0357 (9)	0.0175 (8)	−0.0171 (9)
N1	0.0216 (8)	0.0245 (8)	0.0304 (8)	−0.0099 (6)	0.0014 (7)	−0.0087 (7)
N2	0.0224 (8)	0.0255 (8)	0.0276 (8)	−0.0119 (7)	0.0019 (6)	−0.0073 (7)
N3	0.0196 (8)	0.0236 (8)	0.0301 (8)	−0.0089 (6)	0.0003 (6)	−0.0082 (7)
N4	0.0279 (9)	0.0267 (9)	0.0437 (11)	−0.0140 (7)	0.0035 (8)	−0.0074 (8)
C1	0.0182 (9)	0.0219 (9)	0.0274 (9)	−0.0062 (7)	−0.0007 (7)	−0.0063 (7)
C2	0.0187 (9)	0.0224 (9)	0.0306 (10)	−0.0058 (7)	0.0013 (7)	−0.0053 (8)
C3	0.0170 (9)	0.0210 (9)	0.0334 (10)	−0.0066 (7)	0.0015 (7)	−0.0033 (8)
C4	0.0216 (9)	0.0211 (9)	0.0344 (10)	−0.0091 (7)	−0.0002 (8)	−0.0066 (8)
C5	0.0202 (9)	0.0219 (9)	0.0266 (9)	−0.0080 (7)	−0.0005 (7)	−0.0059 (7)
C6	0.0194 (9)	0.0228 (9)	0.0263 (9)	−0.0072 (7)	−0.0020 (7)	−0.0049 (7)
C7	0.0188 (9)	0.0211 (9)	0.0274 (9)	−0.0065 (7)	−0.0014 (7)	−0.0044 (7)
C8	0.0218 (9)	0.0252 (9)	0.0322 (10)	−0.0091 (8)	0.0033 (8)	−0.0088 (8)
C9	0.0232 (10)	0.0243 (9)	0.0366 (11)	−0.0095 (8)	0.0006 (8)	−0.0114 (8)
C10	0.0207 (9)	0.0214 (9)	0.0340 (10)	−0.0098 (7)	0.0004 (8)	−0.0039 (8)
C11	0.0231 (10)	0.0302 (10)	0.0292 (10)	−0.0108 (8)	0.0020 (8)	−0.0050 (8)
C12	0.0245 (10)	0.0275 (10)	0.0274 (10)	−0.0109 (8)	−0.0010 (8)	−0.0070 (8)
C13	0.0288 (11)	0.0302 (10)	0.0292 (10)	−0.0116 (8)	0.0019 (8)	−0.0129 (8)
C14	0.0322 (11)	0.0288 (10)	0.0312 (10)	−0.0111 (9)	0.0041 (8)	−0.0073 (9)
C15	0.0383 (13)	0.0298 (11)	0.0386 (12)	−0.0096 (9)	0.0092 (10)	−0.0117 (9)
C16	0.0457 (14)	0.0465 (14)	0.0412 (13)	−0.0219 (12)	0.0138 (11)	−0.0168 (11)
C17	0.0528 (16)	0.0397 (14)	0.0486 (15)	−0.0129 (12)	0.0184 (12)	−0.0102 (11)
C18	0.0584 (17)	0.0536 (16)	0.0413 (14)	−0.0229 (14)	0.0172 (12)	−0.0148 (12)
C19	0.0665 (19)	0.0431 (15)	0.0488 (15)	−0.0142 (13)	0.0258 (14)	−0.0070 (12)
C20	0.0589 (18)	0.0568 (17)	0.0365 (13)	−0.0198 (14)	0.0158 (12)	−0.0089 (12)
C21	0.0590 (17)	0.0378 (14)	0.0466 (15)	−0.0096 (12)	0.0208 (13)	−0.0058 (11)
C22	0.0490 (15)	0.0473 (14)	0.0335 (12)	−0.0146 (12)	0.0101 (11)	−0.0101 (11)
C23	0.0486 (15)	0.0449 (14)	0.0370 (12)	−0.0135 (12)	0.0122 (11)	−0.0129 (11)
C24	0.0434 (14)	0.0483 (14)	0.0307 (11)	−0.0158 (11)	0.0009 (10)	−0.0069 (10)
C24A	0.0434 (14)	0.0483 (14)	0.0307 (11)	−0.0158 (11)	0.0009 (10)	−0.0069 (10)

### Geometric parameters (Å, º)

**Table:**

Br1—C3	1.8954 (19)	C14—H14A	0.9900
Br2—C24	1.953 (3)	C14—H14B	0.9900
Br2A—C24A	1.962 (5)	C15—C16	1.520 (3)
O1—N4	1.224 (2)	C15—H15A	0.9900
O2—N4	1.233 (2)	C15—H15B	0.9900
N1—C5	1.357 (2)	C16—C17	1.514 (3)
N1—C4	1.361 (2)	C16—H16A	0.9900
N1—C13	1.473 (3)	C16—H16B	0.9900
N2—C5	1.336 (2)	C17—C18	1.526 (4)
N2—C6	1.372 (3)	C17—H17A	0.9900
N3—C6	1.346 (2)	C17—H17B	0.9900
N3—C1	1.372 (2)	C18—C19	1.511 (4)
N4—C10	1.465 (2)	C18—H18A	0.9900
C1—C2	1.392 (3)	C18—H18B	0.9900
C1—C5	1.423 (3)	C19—C20	1.520 (4)
C2—C3	1.396 (3)	C19—H19A	0.9900
C2—H2	0.9500	C19—H19B	0.9900
C3—C4	1.376 (3)	C20—C21	1.521 (4)
C4—H4	0.9500	C20—H20A	0.9900
C6—C7	1.467 (3)	C20—H20B	0.9900
C7—C8	1.393 (3)	C21—C22	1.517 (4)
C7—C12	1.400 (3)	C21—H21A	0.9900
C8—C9	1.383 (3)	C21—H21B	0.9900
C8—H8	0.9500	C22—C23	1.526 (3)
C9—C10	1.387 (3)	C22—H22A	0.9900
C9—H9	0.9500	C22—H22B	0.9900
C10—C11	1.380 (3)	C23—C24A	1.507 (3)
C11—C12	1.389 (3)	C23—C24	1.507 (3)
C11—H11	0.9500	C23—H23A	0.9900
C12—H12	0.9500	C23—H23B	0.9900
C13—C14	1.521 (3)	C24—H24A	0.9900
C13—H13A	0.9900	C24—H24B	0.9900
C13—H13B	0.9900	C24A—H24C	0.9900
C14—C15	1.531 (3)	C24A—H24D	0.9900
			
C5—N1—C4	118.57 (17)	C17—C16—C15	115.0 (2)
C5—N1—C13	119.67 (16)	C17—C16—H16A	108.5
C4—N1—C13	121.75 (17)	C15—C16—H16A	108.5
C5—N2—C6	100.79 (16)	C17—C16—H16B	108.5
C6—N3—C1	102.17 (16)	C15—C16—H16B	108.5
O1—N4—O2	123.30 (19)	H16A—C16—H16B	107.5
O1—N4—C10	118.48 (18)	C16—C17—C18	113.4 (2)
O2—N4—C10	118.22 (18)	C16—C17—H17A	108.9
N3—C1—C2	132.04 (18)	C18—C17—H17A	108.9
N3—C1—C5	107.59 (16)	C16—C17—H17B	108.9
C2—C1—C5	120.37 (18)	C18—C17—H17B	108.9
C1—C2—C3	115.51 (18)	H17A—C17—H17B	107.7
C1—C2—H2	122.2	C19—C18—C17	115.1 (2)
C3—C2—H2	122.2	C19—C18—H18A	108.5
C4—C3—C2	123.24 (18)	C17—C18—H18A	108.5
C4—C3—Br1	115.85 (15)	C19—C18—H18B	108.5
C2—C3—Br1	120.92 (15)	C17—C18—H18B	108.5
N1—C4—C3	120.73 (18)	H18A—C18—H18B	107.5
N1—C4—H4	119.6	C18—C19—C20	113.3 (3)
C3—C4—H4	119.6	C18—C19—H19A	108.9
N2—C5—N1	126.94 (18)	C20—C19—H19A	108.9
N2—C5—C1	111.52 (17)	C18—C19—H19B	108.9
N1—C5—C1	121.53 (17)	C20—C19—H19B	108.9
N3—C6—N2	117.92 (17)	H19A—C19—H19B	107.7
N3—C6—C7	121.76 (17)	C19—C20—C21	114.2 (3)
N2—C6—C7	120.31 (17)	C19—C20—H20A	108.7
C8—C7—C12	119.67 (18)	C21—C20—H20A	108.7
C8—C7—C6	120.18 (18)	C19—C20—H20B	108.7
C12—C7—C6	120.14 (18)	C21—C20—H20B	108.7
C9—C8—C7	120.52 (19)	H20A—C20—H20B	107.6
C9—C8—H8	119.7	C22—C21—C20	113.0 (2)
C7—C8—H8	119.7	C22—C21—H21A	109.0
C8—C9—C10	118.46 (19)	C20—C21—H21A	109.0
C8—C9—H9	120.8	C22—C21—H21B	109.0
C10—C9—H9	120.8	C20—C21—H21B	109.0
C11—C10—C9	122.63 (18)	H21A—C21—H21B	107.8
C11—C10—N4	119.21 (18)	C21—C22—C23	112.7 (2)
C9—C10—N4	118.16 (18)	C21—C22—H22A	109.0
C10—C11—C12	118.37 (19)	C23—C22—H22A	109.0
C10—C11—H11	120.8	C21—C22—H22B	109.0
C12—C11—H11	120.8	C23—C22—H22B	109.0
C11—C12—C7	120.34 (19)	H22A—C22—H22B	107.8
C11—C12—H12	119.8	C24A—C23—C22	114.3 (2)
C7—C12—H12	119.8	C24—C23—C22	114.3 (2)
N1—C13—C14	112.14 (17)	C24—C23—H23A	108.7
N1—C13—H13A	109.2	C22—C23—H23A	108.7
C14—C13—H13A	109.2	C24—C23—H23B	108.7
N1—C13—H13B	109.2	C22—C23—H23B	108.7
C14—C13—H13B	109.2	H23A—C23—H23B	107.6
H13A—C13—H13B	107.9	C23—C24—Br2	114.01 (18)
C13—C14—C15	111.20 (18)	C23—C24—H24A	108.8
C13—C14—H14A	109.4	Br2—C24—H24A	108.8
C15—C14—H14A	109.4	C23—C24—H24B	108.8
C13—C14—H14B	109.4	Br2—C24—H24B	108.8
C15—C14—H14B	109.4	H24A—C24—H24B	107.6
H14A—C14—H14B	108.0	C23—C24A—Br2A	99.5 (3)
C16—C15—C14	113.51 (19)	C23—C24A—H24C	111.9
C16—C15—H15A	108.9	Br2A—C24A—H24C	111.9
C14—C15—H15A	108.9	C23—C24A—H24D	111.9
C16—C15—H15B	108.9	Br2A—C24A—H24D	111.9
C14—C15—H15B	108.9	H24C—C24A—H24D	109.6
H15A—C15—H15B	107.7		
			
C6—N3—C1—C2	−179.2 (2)	C6—C7—C8—C9	−179.63 (18)
C6—N3—C1—C5	0.9 (2)	C7—C8—C9—C10	−0.5 (3)
N3—C1—C2—C3	179.6 (2)	C8—C9—C10—C11	1.5 (3)
C5—C1—C2—C3	−0.4 (3)	C8—C9—C10—N4	−178.13 (18)
C1—C2—C3—C4	2.3 (3)	O1—N4—C10—C11	176.3 (2)
C1—C2—C3—Br1	−177.37 (13)	O2—N4—C10—C11	−3.8 (3)
C5—N1—C4—C3	−0.3 (3)	O1—N4—C10—C9	−4.1 (3)
C13—N1—C4—C3	−179.31 (18)	O2—N4—C10—C9	175.81 (19)
C2—C3—C4—N1	−2.0 (3)	C9—C10—C11—C12	−1.0 (3)
Br1—C3—C4—N1	177.68 (14)	N4—C10—C11—C12	178.54 (18)
C6—N2—C5—N1	−178.66 (19)	C10—C11—C12—C7	−0.3 (3)
C6—N2—C5—C1	−0.1 (2)	C8—C7—C12—C11	1.2 (3)
C4—N1—C5—N2	−179.38 (18)	C6—C7—C12—C11	−179.94 (18)
C13—N1—C5—N2	−0.4 (3)	C5—N1—C13—C14	−83.3 (2)
C4—N1—C5—C1	2.2 (3)	C4—N1—C13—C14	95.7 (2)
C13—N1—C5—C1	−178.80 (18)	N1—C13—C14—C15	169.76 (17)
N3—C1—C5—N2	−0.5 (2)	C13—C14—C15—C16	−74.4 (3)
C2—C1—C5—N2	179.53 (17)	C14—C15—C16—C17	−168.7 (2)
N3—C1—C5—N1	178.14 (17)	C15—C16—C17—C18	177.0 (2)
C2—C1—C5—N1	−1.8 (3)	C16—C17—C18—C19	−179.0 (3)
C1—N3—C6—N2	−1.0 (2)	C17—C18—C19—C20	178.2 (3)
C1—N3—C6—C7	178.18 (17)	C18—C19—C20—C21	175.8 (3)
C5—N2—C6—N3	0.7 (2)	C19—C20—C21—C22	−173.9 (3)
C5—N2—C6—C7	−178.50 (17)	C20—C21—C22—C23	172.0 (3)
N3—C6—C7—C8	−0.5 (3)	C21—C22—C23—C24A	−169.6 (2)
N2—C6—C7—C8	178.74 (18)	C21—C22—C23—C24	−169.6 (2)
N3—C6—C7—C12	−179.32 (18)	C22—C23—C24—Br2	−66.7 (3)
N2—C6—C7—C12	−0.1 (3)	C22—C23—C24A—Br2A	−76.3 (3)
C12—C7—C8—C9	−0.8 (3)		

### Hydrogen-bond geometry (Å, º)

**Table:**

*D*—H···*A*	*D*—H	H···*A*	*D*···*A*	*D*—H···*A*
C4—H4···O1^i^	0.95	2.43	3.171 (3)	135
C24—H24*A*···O2^ii^	0.99	2.53	3.372 (3)	142

Symmetry codes: (i) *x*+1, *y*+1, *z*; (ii) −*x*, −*y*+1, −*z*+1.
